# Correction: Roles for TAB1 in regulating the IL-1-dependent phosphorylation of the TAB3 regulatory subunit and activity of the TAK1 complex

**DOI:** 10.1042/BJ20071149_COR

**Published:** 2025-06-18

**Keywords:** interleukin-1 (IL-1), NF-κB, TAB1, TAK1, TNFα

It has come to the attention of the authors of the article “Roles for TAB1 in regulating the IL-1-dependent phosphorylation of the TAB2 regulatory subunit and activity of the TAK1 complex” (DOI: 10.1042/BJ20071149) that there is a duplication within Figure 3 – namely that the same data has been used to represent Total TAB2 in Figure 3.

**Figure 3:**
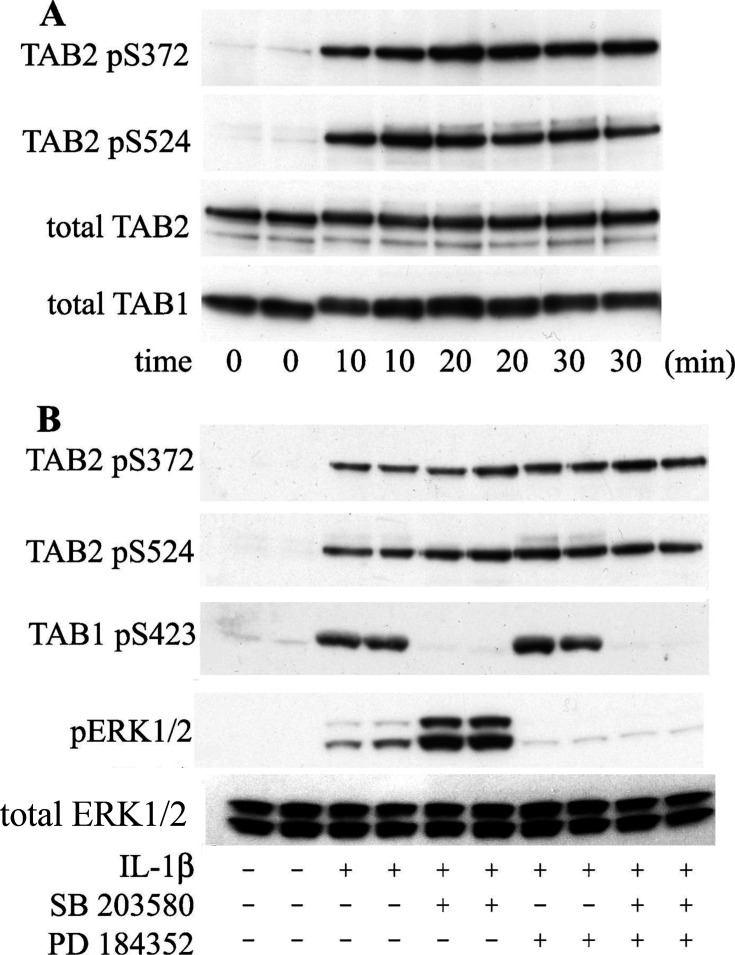
Effect of IL-1β on the site-specific phosphorylation of TAB2. (**A**) IL-1R cells were serum-starved for 6 h, then stimulated for the times indicated with IL-1β (5 ng/ml) and lysed. The TAK1 complex was immunoprecipitated from the cell extracts (2 mg of extract protein) using an anti-TAB1 antibody, denatured in LDS (lithium dodecyl sulfate), subjected to SDS/PAGE and immunoblotted with phospho-specific antibodies that recognize the phosphorylated residues indicated and with further antibodies that recognize the TAB2 and TAB1 proteins. (**B**) As in (**A**), except that, after serum deprivation, the cells were incubated for 1 h in the presence (+) or absence (-) of 5 µM SB 203580 and/or 2 µM PD 184352 before stimulation for 20 min with (+) or without (-) IL-1β. Further aliquots of the cell extracts from (**B**) were immunoblotted (without immunoprecipitation) using antibodies that recognize TAB1 phosphorylated at Ser423, the active phosphorylated forms of ERK1/2 and total ERK1/2.

Original data for this experiment have been assessed, and it has been confirmed that the Total TAB2 image was correctly used in Figure 3A and was mistakenly included in Figure 3B. Figure 3B includes key controls with the inclusion of the immunoblots for phospho-TAB1 and phospho-ERK1/2. A further control was performed using total ERK1/2, and this has been included in a revised figure presented here.

The requested correction has been assessed by and agreed with the Publisher. The authors apologise for the error and any inconvenience this may have caused. The data analysis and conclusions are not affected by the error.

A corrected Figure 3 is presented here.

